# Factors influencing the uptake of antenatal care in Uganda: a mixed methods systematic review

**DOI:** 10.1186/s12884-024-06938-6

**Published:** 2024-11-08

**Authors:** Kiran Bhutada, Mahima Venkateswaran, Maureen Atim, Susan Munabi-Babigumira, Victoria Nankabirwa, Flavia Namagembe, J. Frederik Frøen, Eleni Papadopoulou

**Affiliations:** 1 Global Health Cluster, Division for Health Services, Norwegian Institute of Public Health, PO Box 222, Skøyen, Oslo Norway; 2 Global Health Center, Albert Einstein College of Medicine, 1300 Morris Park Avenue Block 505, Bronx, NY 10461 USA; 3 Centre for Intervention Science in Maternal and Child Health (CISMAC), University of Bergen, Bergen, Norway; 4 Makerere University School of Public Health, New Mulago Gate Rd, Kampala, Uganda; 5 Department of Health and Functioning, Western Norway University of Applied Sciences, Bergen, Norway

**Keywords:** Antenatal care, Healthcare utilization, Respectful care, Delivery of health care, Maternity care, ANC guidelines, Health workers, Uganda, Barriers, Implementation

## Abstract

**Background:**

In 2016, the World Health Organization (WHO) recommended increasing antenatal care (ANC) visits from four to eight to reduce maternal morbidity and mortality. However, many low-middle income countries (LMICs), including Uganda, struggle to achieve even the basic four ANC visits. To further improve ANC attendance, understanding the perceptions and beliefs of end users is crucial. This systematic review explores the perceptions, experiences, and behavior of pregnant or previously pregnant women, their families and healthcare workers on ANC attendance in Uganda.

**Methods:**

The review includes qualitative and quantitative studies published from January 2012 to September 2022. Outcomes include early initiation of ANC visits and any attendance or utilization of routine ANC services. The Critical Appraisal Skills Programme (CASP) checklist was used to assess the quality of included studies.

**Results:**

We searched 7 databases, identified 725 references and assessed 107 in full text for eligibility based on selected inclusion criteria. Forty-seven studies were eligible and are included in this review. Quantitative findings highlight socioeconomic factors like occupation, wealth index, and marital status as key determinants of ANC uptake and timely uptake of care, favoring higher wealth, younger age, marriage, and media access. Qualitative evidence reveals challenges to ANC attendance including financial constraints, cultural beliefs, gendered decision-making, and geographical distance from healthcare facilities. Potential solutions involve financially empowering women, providing reliable ANC equipment and medication, and community engagement and education.

**Conclusions:**

This review offers valuable insights for policymakers and healthcare providers seeking to tailor interventions that address the unique needs and challenges faced by pregnant women, their families, and healthcare workers in Uganda. By doing so, it may enhance ANC accessibility and quality, ultimately aligning with the WHO’s recommendation of eight ANC contacts (ANC8) and contributing to reducing maternal morbidity and mortality rates.

**Supplementary Information:**

The online version contains supplementary material available at 10.1186/s12884-024-06938-6.

## Background

Globally, about 295,000 women die during childbirth every year, with more than 90% of the deaths happening in low- and middle-income countries (LMIC) [1]. Sub-Saharan Africa accounts for the biggest burden (66%) of maternal deaths. In Uganda, the pregnancy-related mortality ratio is 228 deaths per 100,000 live births [2] and for every accounted maternal death, 20 to 30 more women suffer from childbirth related injuries, infections, or disabilities [3]. Antenatal care (ANC), in addition to healthcare during delivery, and the postpartum period, is crucial for reducing maternal mortality and morbidity.

In 2016, The World Health Organization (WHO) revised its recommendation for the number of ANC visits from four visits to eight contacts [4] (ANC8). These contacts offer women prenatal counseling, nutrition advice, and an opportunity to detect and manage potential complications. In Uganda, free ANC services are provided at public health facilities. The 2022 Ugandan Demographic Health Survey (DHS) found that 95% of pregnant women received ANC from a skilled health professional at least once, about 72% of women attended ANC at least four times during their pregnancy [2]. Contrastingly, the 2016 UDHS reported that only 30% of these women initiated ANC in the first trimester [5, 6]. Timeliness (attending ANC at designated times in pregnancy), quantity, and quality of visits remain a concern.

The WHO has identified numerous obstacles to effectively implementing the recommended eight ANC visits [1, 4]. These challenges encompass a scarcity of skilled human resources, deficient infrastructure, limited physical space, inadequate medical supplies, healthcare worker comprehension issues, community misconceptions, and insufficient health information management systems (HMISs) [4, 7]. With the introduction of eight ANC contacts, such barriers are only increased, and implementation made more challenging. Furthermore, previous research has highlighted the hurdles that women and their families encounter, globally, when seeking access to ANC [7–9]. Factors such as socioeconomic disparities, substandard infrastructure, resource insufficiency, and transportation difficulties prominently hinder ANC service utilization, particularly for those residing in rural areas [4]. Healthcare workers (HCW) have similarly described insufficient medical equipment and infrastructure, a lack of health care personnel, and timing constraints as impediments to high quality ANC [8]. Beyond these challenges, a dearth of information, cultural beliefs, gender norms, insufficient government funding, and inadequate political prioritization of ANC services collectively curtail the availability and effectiveness of maternal healthcare, including ANC [8].

The purpose of this systematic review was to examine the perspectives, values, preferences, and satisfaction of women who are or have received ANC in Uganda. Additionally, the viewpoints of their families and HCWs were included in this investigation. The review focused on exploring factors that impede or enable utilization of ANC services, with an additional aim to identify and inform innovations for the implementation of ANC8 in Uganda.

## Methods

### Study inclusion and exclusion criteria

We included both qualitative and quantitative primary studies published within the past ten years (since 2012). Articles included in the review evaluated the perceptions and beliefs of pregnant or previously pregnant women, their families, and healthcare workers on the provision of ANC in Uganda. The qualitative studies generally aimed to encompass the complex experiences, intersecting beliefs, and perceptions of ANC in the Ugandan context typically using ethnography or phenomenology. Other designs eligible for review included case series, and mixed method designs. Data collection methods included interviews, focus groups, open ended survey questions, and observations. Studies conducted using qualitative methods but without thorough qualitative analysis were not eligible for inclusion.

We also included quantitative study designs to identify separate or similar themes. Data collection methods included survey questionnaires, such as secondary analysis of DHS, and healthcare checklists. Mixed method studies were also included. We excluded intervention studies, as their scope is outside our study aim. Conference abstracts were also excluded due to insufficient information.

### Types of participants

This review included studies focused on three key stakeholder groups: pregnant or previously pregnant women, family members of these women, and healthcare workers involved in the provision of ANC in Uganda. We included studies that involved currently pregnant women or women who were pregnant at some time since 2012, their family members such as partners and parents, or HCWs. Studies included participants in any setting where ANC was provided including, but not limited to, outpatient/ANC clinics, antenatal, delivery and postnatal wards in hospitals, birth centers, women’s homes, and electronic or m-health platforms. Studies of women who had not given birth in the past ten years or were not currently pregnant were excluded.

We included studies that reported the views and experiences of any healthcare provider (doctors, nurses, lay health workers, pharmacists, student healthcare workers providing care as part of training, or other healthcare workers providing home-based or community-based ANC [8]) involved in ANC provision. We did not impose any restriction on healthcare providers in the study selection, and we included healthcare workers in government health facilities (Level I, II, III, IV, and Hospital workers), private clinics, those working under charity funders, community health workers, and traditional birthing assistants (TBAs).

### Types of interventions and outcome measures

Interventions of interest included all those related to routine ANC. Routine ANC was understood as a range of care services that all pregnant women have access to, including counseling, tests, treatment, health promotion activities, information, and any supportive measures. Our definition of ANC was not limited to specific contacts or visits, but rather encompassed all the care and support women receive during pregnancy. Studies reporting the impact of such interventions on family members and healthcare workers were included. We excluded studies focused on birth and delivery care, postnatal care, and postnatal interventions only. We also excluded studies focused on neonatal outcomes without reported assessments of ANC.

The primary phenomenon of interest were the perspectives, values, and behaviors towards ANC from the viewpoint of three groups of participants, and how these influence the uptake and provision of routine ANC services. Secondary phenomenon included early factors influencing initiation of ANC, any ANC attendance, retention at ANC, satisfaction and preferences, sustainability, willingness to participate in ANC, feasibility, views on quality of care, and importance of care.

### Search methods

The search strategy was designed to access published and unpublished studies. We searched the following electronic databases: Medline, Cinahl, EMBASE, Epistemonikos, Global Index Medicus, AFROLIB.

We developed search strategies for each database (Additional file 1). A geographic limit was set to focus results to Uganda. We did not impose any language restrictions. To encapsulate relevant views and experiences pertaining to recent and current practices in ANC, we limited our publication year to 2012 and onward. The search took place between September 6th and 8th, 2022. Hand searching was done via the Makerere University Undergraduate Dissertation Repository [10] and published systematic reviews [8, 11, 12].

### Data extraction and analysis

All references were uploaded into Covidence, a web-based platform for systematic literature reviews [13]. Duplicate studies were removed. One review author (KB) assessed each study based on title and abstract against pre-decided inclusion and exclusion criteria. Following this phase, two review authors (KB and MA) reviewed the full text of all remaining articles against the inclusion and exclusion criteria. For studies in conflict, a third reviewer (EP) conducted an independent assessment. A final decision was made by consensus of all three reviewers. Reasons for exclusion were recorded. All studies retrieved were in English, so no translations were necessary. None of the review authors were involved in the conduct, analysis, and publication of a study that could be included in the review.

Two reviewers (KB and EP) conducted data extraction for both qualitative and quantitative studies. Regular meetings of the reviewers were held to resolve any discrepancies or uncertainties in the extracted data. The content of extracted items from the studies was decided based on discussion among the team and previous systematic reviews on the subject [8, 11, 12]. For qualitative studies, direct quotes from participants were also extracted. For quantitative studies, relevant numerical data were extracted directly into Excel.

Through an iterative process, qualitative data were coded and categorized based on key themes from the perspective of all three populations studied. We then compiled the major themes based on a synthesis and translation of categorized data, following the principles of thematic synthesis [14]. As each full text study was read, codes, themes, and analysis from original authors were extracted along with verbatim text. This facilitated initial marking of factors as either barrier or enabler to ANC access, suggested classification of quality of care, and potential societal impacts. As data were extracted, a list of thematic content was formulated, added to, and revised. With the last study extraction, this list was finalized. Analysis continued through summaries of key themes.

For quantitative studies, we extracted the data using a standardized data collection form. Extracted data were coded consistently to support further data synthesis. Given the exploratory research question of our review, we did not meta-analyze the extracted data, and rather reported them narratively. We used plots to visualize the findings of each included study. To understand the importance of each included factor, we extracted data only from studies reporting multivariate adjusted associations. We extracted regression coefficients for the studied determinants of ANC quality outcomes from 11 out of 15 quantitative studies which used logistic regression, while four [15–18] used a different statistical methodology or did not report regression coefficients.

### Assessing the methodological limitations of included studies

For qualitative studies, one reviewer (KB) followed the Critical Appraisal Skills Programme (CASP) [19] checklist to ensure study quality, based on the following: (A) Are the results of the study valid? (B) What are the results? (C) Will the results help locally? This checklist allows researchers to consider such issues systematically. We assessed the research aims, methodology, research design, recruitment strategy, data collection methods, participant-researcher relationship, ethical considerations, data analysis, findings, and value of research. Studies with a score of 3 or more “no’s” or “can’t tell” were deemed to have methodological limitations (Additional file 2). We did not assess the methodological quality of quantitative studies for pragmatic reasons given the large number of DHS studies (10 out of 15 quantitative studies).

### Review of author reflexivity

To enhance the trustworthiness and rigor of the review, we engaged in several methods of author reflexivity [20]. The authors examined their personal outlooks on the decisions surrounding the emergence of key themes, study findings, and overall review results. The research team also engaged in discussion sessions to reflect on experiences and insights brought into this work. All authors of this review had the belief that ANC, both formal and informal, is valuable to the health and well-being of women. Satisfaction and access to this care through the lens of women was emphasized, including the idea of provision of ANC as more than medical care, but also the psychosocial responsibilities of HCWs and support systems. Throughout data extraction and analysis, iterative processes were undertaken to ensure reliable synthesis of findings.

## Results

**Fig. 1:**
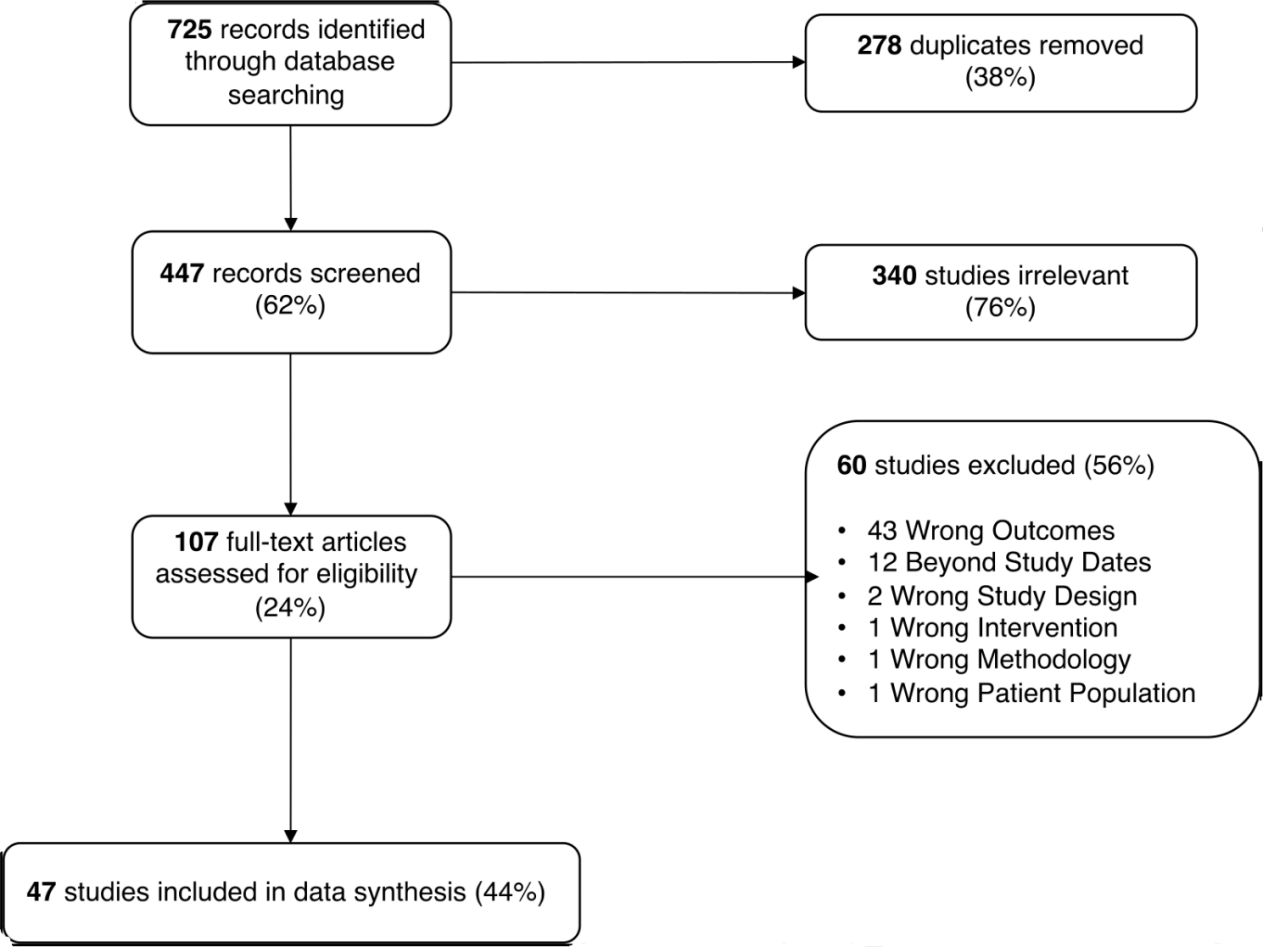
PRISMA diagram illustrating the search result process

Our search identified 725 records. 447 records were remaining following duplicate exclusion (Fig. 1). After title and abstract screening, we retrieved 107 full-text articles which were assessed for eligibility. Of these, 60 studies were excluded: 43 for wrong outcomes, 12 published before 2012, and 5 others for wrong study design, intervention, methodology and patient population. After the full-text review, 47 studies published from January 2012 to September 2022 were included in this review (Additional file 3). All studies were published in English.

The studies focused on women living in rural, urban, and semi-urban areas in Uganda. Studies included women who had received ANC in health facilities and at home, as well as women who received care from an unofficial provider or defaulted from care. Overall, 17 (36%) of 47 studies were conducted with qualitative methodology only. Methods included grounded theory, phenomenology, individual and key-informant interviews, and focus group discussions. In contrast, 15 (32%) of 47 studies included quantitative methodology only, the majority involving DHS surveys (10 out of 15). The remaining 15 (32%) studies were mixed, including both qualitative and quantitative methods. Only one of the studies with mixed methods had eligible quantitative data for extraction, while all 15 were included in the qualitative data extraction. Overall, quantitative data were extracted from 12 studies and qualitative data from 32 studies.

The majority (87%, *n* = 41) of included literature studied the population of pregnant or previously pregnant women, 53% involved HCWs, and 26% included family members. Many studies had mixed populations (Table 1). Of 47 studies, 37 were addressing barriers and enablers to ANC broadly. 4 papers more specifically discussed the ramifications of gender on ANC access, 2 papers discussed the impact of the COVID-19 pandemic on ANC, 2 focused on ANC for indigenous women, and one each studied the effect of media access and war on ANC attendance.

**Table 1:** Characteristics of the 47 included studies

Characteristics	Number	Percentage
**Geographic location**		
Uganda only	36	77%
Uganda and other countries*	11	23%
**Region**		
Rural	27	58%
Urban	5	11%
Mixed	15	32%
**Year of Publication**		
2012–2017	29	62%
2018–2022	18	38%
**Study Design**		
Quantitative	15	32%
Qualitative	17	36%
Mixed Methods	15	32%
**Study Population**		
Women Only	16	34%
HCW Only	4	9%
Family Members Only	1	2%
Women, HCW, and Family	6	13%
Women and HCW	14	30%
Women and family	5	11%
HCW and family	1	2%

*Included countries: Bangladesh, Burundi, Cambodia, Cameroon, Ethiopia, Ghana (2), Kenya (2), Malawi, Myanmar, Namibia, Nepal, Nigeria, Peru, Rwanda, Senegal (2), South Africa, Tanzania (3), Zambia; HCW: Healthcare worker

### Quantitative findings

Most quantitative studies in our systematic review primarily examined determinants of ANC uptake or utilization. Nine studies investigated ANC uptake by examining factors related to attending four or more ANC visits, attending any ANC, or achieving optimal ANC attendance [21–29], while five studies focused on ANC utilization by exploring early initiation of ANC [23, 27, 30–32]. These quantitative studies predominantly investigated socioeconomic indicators, including maternal education, occupation, wealth index, marital status, and exposure to media, in descending order of frequency. Obstetric factors such as maternal age, parity, and gravidity, along with demographic factors, particularly region of residence, were also commonly explored. Other socioeconomic factors like partner’s education and occupation, household size, and health insurance, demographic factors like religion and calendar year, variables related to decision-making and sexual empowerment, other obstetric factors (history of pregnancy loss and wanted pregnancy), distance from health facilities, physical burden of healthcare access, community visits, and community visits and dialogues with health workers in pregnancy were less frequently examined.

### Socio-demographic factors: maternal education, occupation, wealth index, marital status, and access to media

**Fig. 2:**
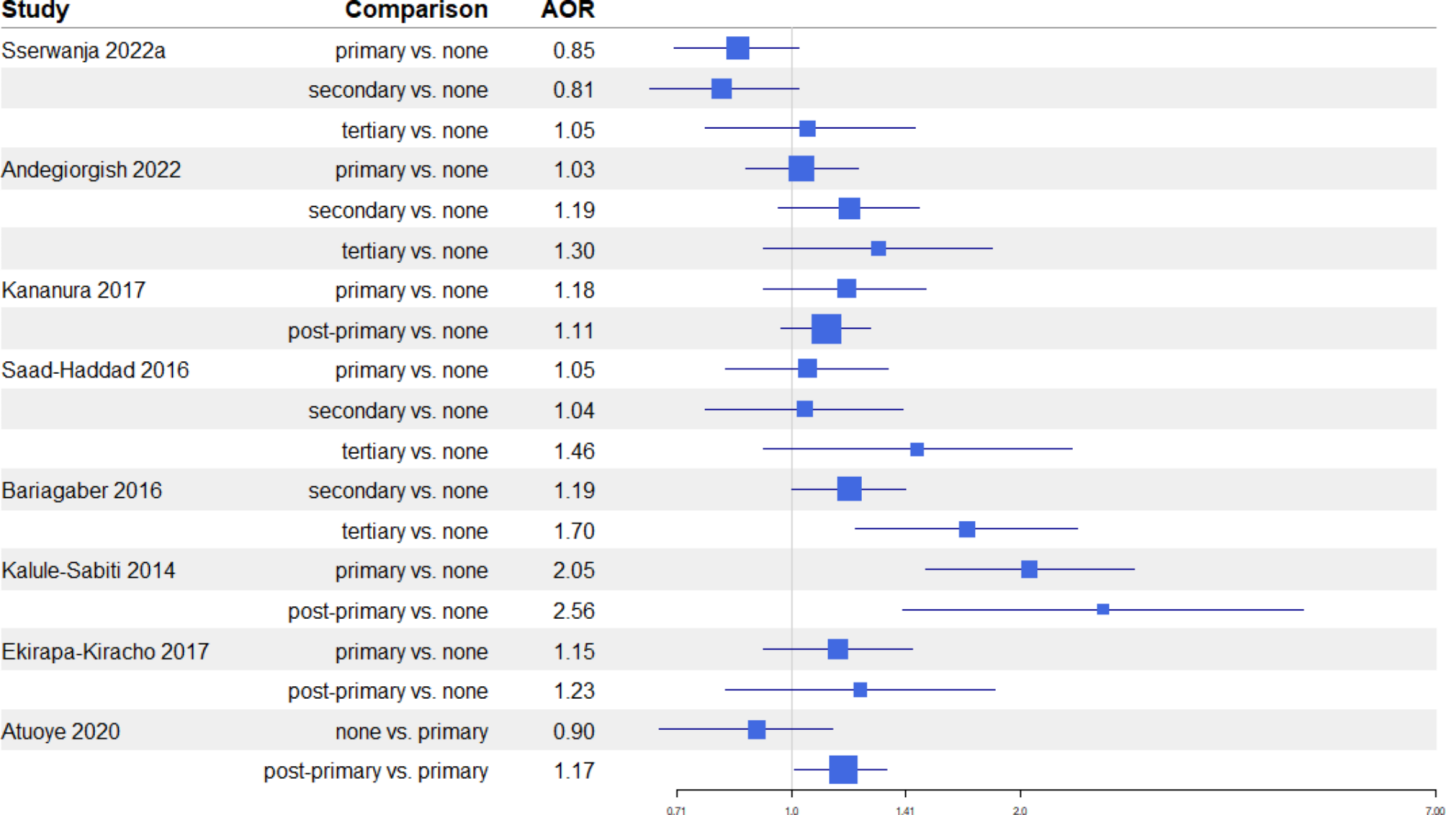
Maternal education and ANC uptake Forest plot showing adjusted Odds Ratio (AOR - squares) and 95% Confidence Intervals (lines) for the associations between maternal educational level and ANC uptake in the included studies

Of the eight studies exploring the association between maternal education and ANC uptake, three reported significant positive associations, favoring higher education levels compared to no or only primary education [23, 25, 26] (Fig. 2). The remaining studies reported similar associations that, while not statistically significant, indicate trends in the same direction [21, 24, 27, 28].

**Fig. 3:**
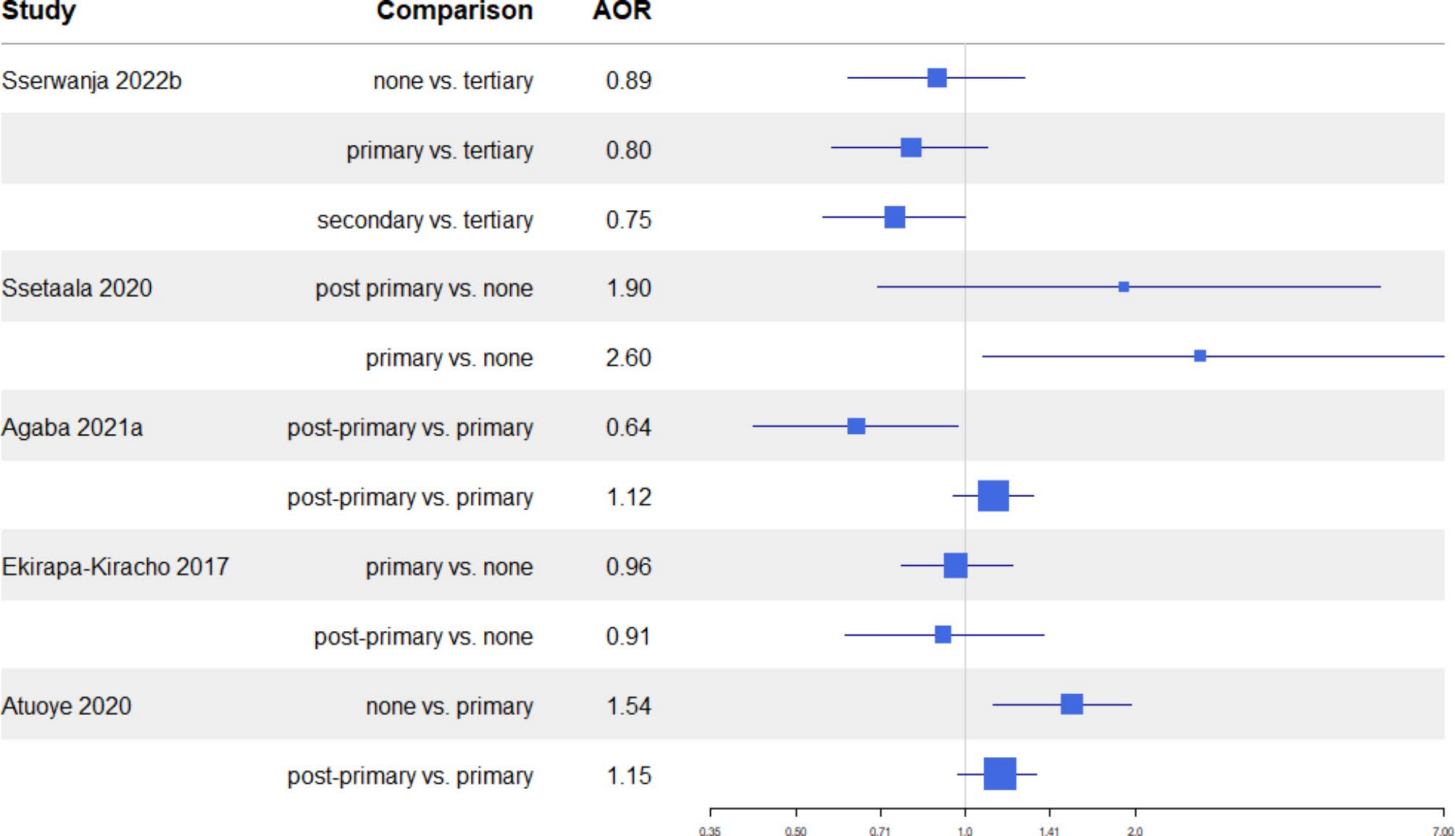
Maternal education and early ANC initiation Forest plot showing adjusted Odds Ratio (AOR - squares) and 95%Confidence Intervals (lines) for the associations between maternal educational level and early ANC initiation in the included studies

Regarding early ANC initiation, among five studies, three reported significant associations between early initiation and education level (Fig. 3). Two indicated an inverse relationship, suggesting a higher likelihood of early initiation with lower education levels [23, 32], while one study found a positive association between primary education and early ANC initiation compared to no education [31]. Other studies suggested a similar inverse relationship, but without statistical significance [27, 30].

Out of seven included studies that reported the association between wealth index and ANC uptake, a positive adjusted association was consistently seen with all but one achieving statistical significance [21, 23–28]. Women in the rich/richest wealth categories exhibited, on average, a 45% higher odds of ANC uptake, with a range spanning from 27 to 98%, when compared to those in the poorest wealth index category [21, 24, 25, 28]. Women in the poorest/poor wealth index level had, on average, a 29% lower odds of ANC uptake, with a range of 15–38%, when compared to those in the richest wealth category [26, 27, 31]. Similarly, regarding ANC utilization, women in the poor/poorest wealth categories showed reduced odds of early ANC initiation compared to those in the rich/richest category [23, 27]. Other wealth-related indicators, such as saving money for ANC, economic empowerment of women, health insurance, and financial burden for ANC, were also studied [23, 27, 28]. Notably, women’s economic empowerment exhibited a positive and significant association with ANC uptake, even after adjusting for various factors, including wealth index and health insurance [28].

Six studies explored the association between maternal occupation and ANC uptake, with most findings lacking significance except one [21, 23, 25, 27, 28]. Andegiorgish AK, et al. reported an increased likelihood of ANC uptake for women in agriculture (AOR = 1.26, 95% CI = 1.09,1.46), professional or skilled workers (AOR = 1.38, 95% CI = 1.18,1.62), and unskilled workers (AOR = 1.62, 95% CI = 1.28,2.05) compared to those without work [21]. Regarding ANC initiation, only one of four studies reported significant results, indicating that being a laborer was associated with a higher likelihood of early ANC initiation compared to being unemployed for unmarried women aged 15–24 years [32]. The variations in definitions, categorizations, and reference groups across these studies complicate direct comparisons of findings.

Among the five studies examining the link between marital status and ANC uptake, only one study by Bariagaber et al. found a significant association, suggesting that being married is associated with a higher odds ratio (OR) for ANC uptake compared to not being married [23, 25, 27, 28]. For early ANC initiation, three studies assessed the relationship with marital status, with one reporting significance [23, 27, 31]. Specifically, Ssetaala et al. reported higher OR for early ANC initiation among women who did not live with their partner compared to those living with their partner (AOR = 2.50, 95% CI = 1.10, 6.00) [31].

Frequent media access was associated with a higher likelihood of ANC uptake, as indicated by three out of four studies examining media access or radio information’s impact on ANC uptake [21, 25, 27, 28]. Likewise, frequent media access was significantly linked to a higher likelihood of early ANC initiation, according to two out of three studies exploring this association [27, 30, 32].

### Demographic and obstetric factors: maternal age, region, and parity

Seven studies examined the association between maternal age and ANC uptake, while four studies investigated its association with early ANC initiation [22, 23, 25–28, 30, 32]. Among these, three studies found that younger age was significantly linked to a higher likelihood of both ANC uptake and early ANC initiation when compared to older age (> 35 years) [27, 28, 30]. In contrast, two other studies reported that older age was significantly associated with a higher likelihood of both ANC uptake and early ANC initiation when compared to younger age (< 20–25 years) [23, 25].

Five studies investigated the relationship between region and ANC uptake as well as early ANC initiation [23, 25, 28, 30, 32]. Being from the west and north regions of Uganda was associated with a higher likelihood of both ANC uptake and early ANC initiation compared to being from the east region [28, 30]. Furthermore, higher AOR for early ANC initiation were reported for women from the north and west regions compared to those from central regions [32]. Conversely, lower AOR for ANC uptake were found for the central, east, and west regions compared to Kampala, the capital of Uganda [25]. Additionally, lower AOR for early ANC initiation were reported for central, east central, east, and west regions compared to the north [23].

Regarding parity or gravidity, six studies examined their association with ANC uptake, with two reporting a significant negative association, indicating a lower likelihood of ANC uptake for women with four or more children compared to those with one child [21, 25]. However, no significant associations were reported between parity and early ANC initiation in the studies included [27, 30, 32].

### Qualitative findings

**Fig. 4:**
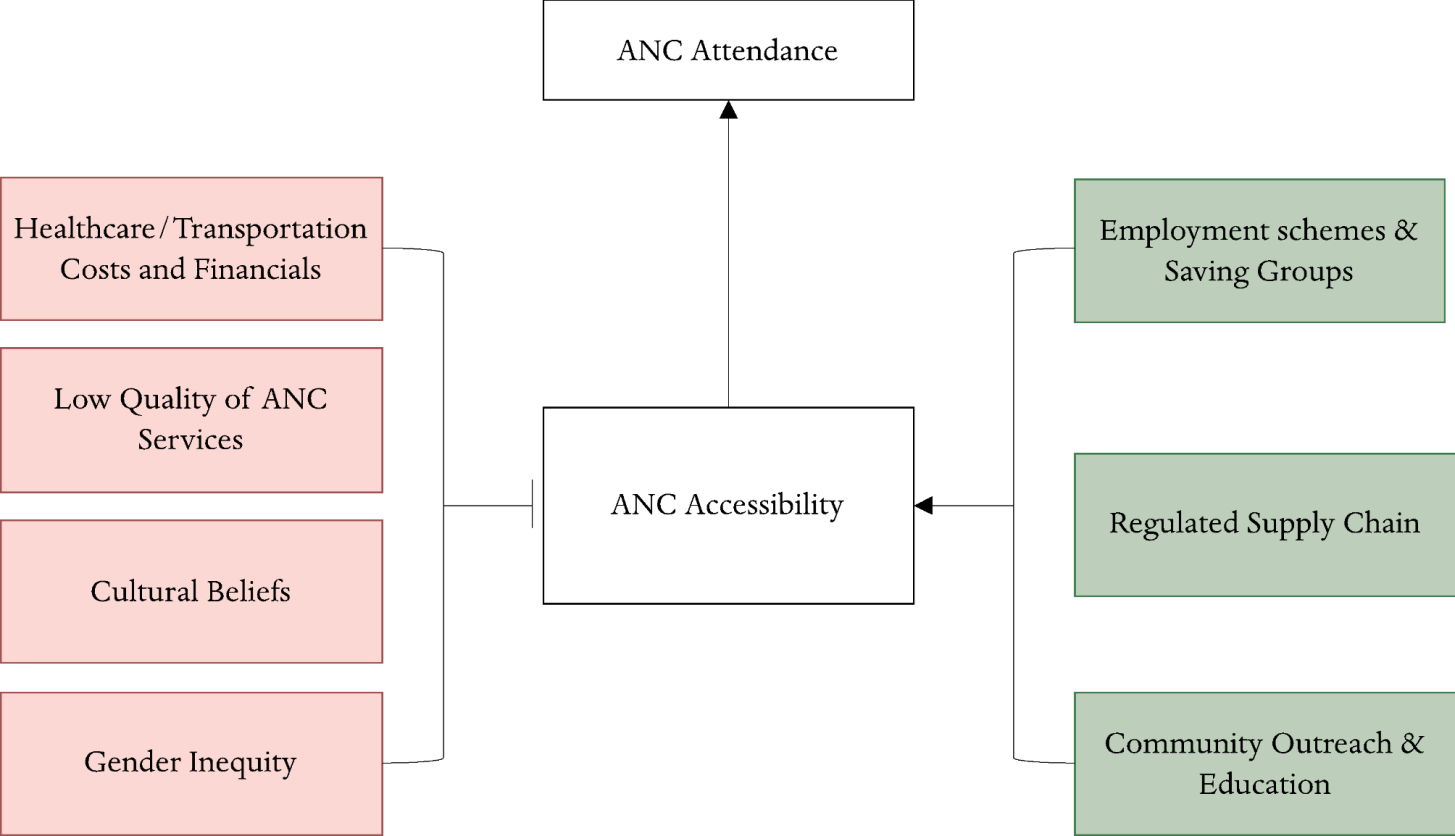
Pathways between barriers and enablers to ANC accessibility and impact on ANC attendance

The findings give us insight into the many perspectives, values, and attitudes of women who have attended ANC, their family members, and HCWs in Uganda. We summarized the main themes from our findings to suggest pathways through various factors influencing ANC accessibility and attendance for these three stakeholders (Fig. 4). Several studies also discuss the enablers of ANC provision in Uganda.

### Barriers to ANC

**Fig. 5:**
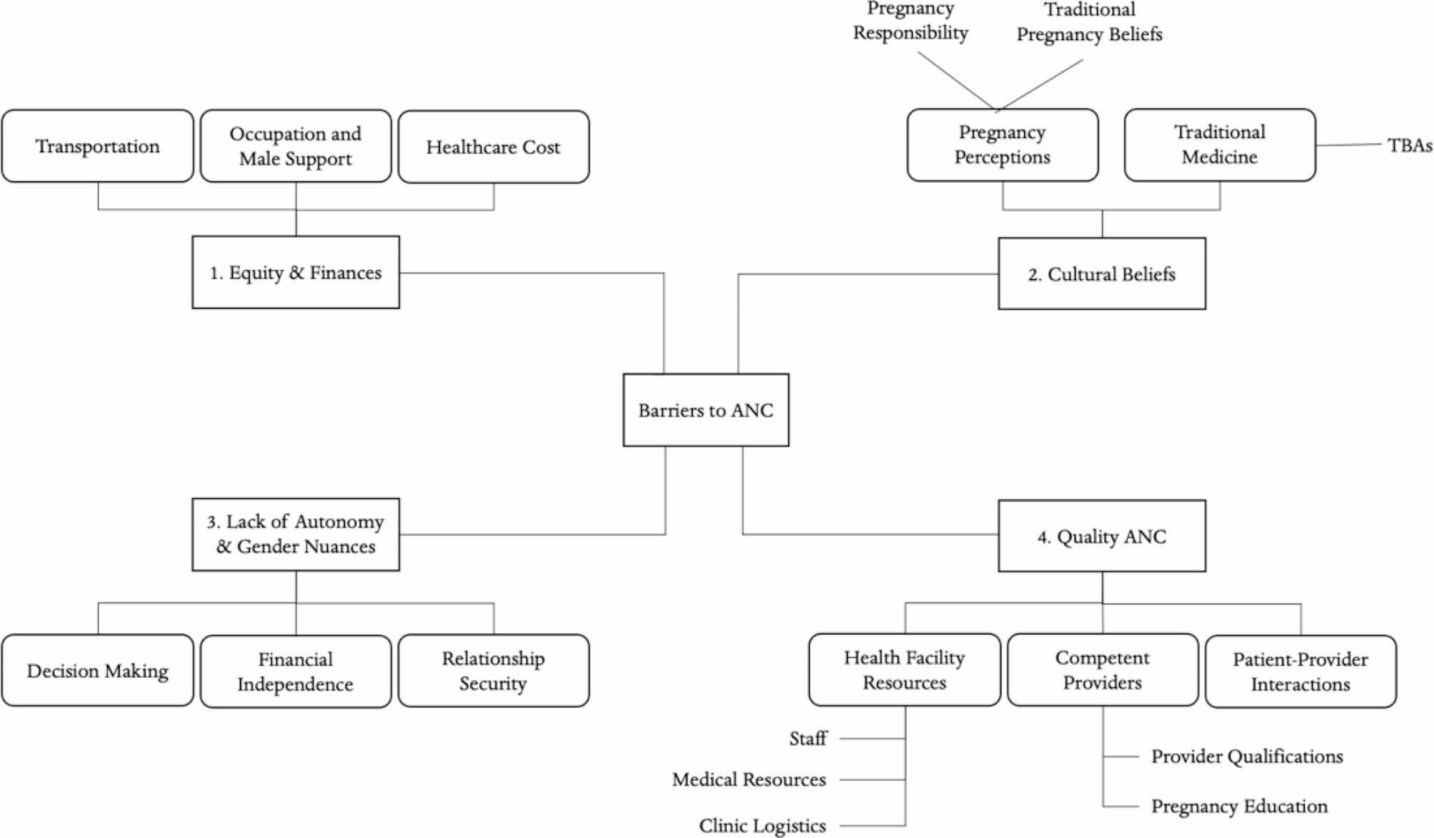
Summary of findings - barriers to attending ANC from women, HCWs, and family members perspective

The literature points to numerous factors that impact women’s access to ANC: finances, cultural beliefs that inhibit early or regular ANC, gendered decision making and autonomy, and low-quality healthcare (Fig. 5).

### Equity and finances

Finances were seen as a large barrier to ANC attendance and accessibility. This included challenges with the cost of transportation, lack of male support, both men and women’s occupation, and the cost of healthcare.

### Transportation

Transportation was cited as a major barrier to ANC accessibility [33–39]. While most women in Uganda attend at least one ANC visit at a health facility [5], the need for repetitive transportation stemmed as a barrier to retention. These perspectives varied by distance to the facility; attending ANC was more feasible for women living closer to health facilities [22, 34, 40, 41]. Women who lived farther from the health facility had to endure expensive and often unsafe transportation, such as motorcycles and “boda-bodas” or walking on possibly dangerous roads [22, 37, 39, 42]. Such travel also depended on road conditions, road traffic, availability of transport, and permission from husbands or partners [22, 35, 43]. The distance to health facilities increased the appeal of visiting TBAs for pregnancy care, as TBAs were often located near their communities [22, 42, 44]. This was especially true for rural women [24]. Women reported their desire to attend ANC but recounted the strain and exhaustion felt from traveling far distances to health facilities [22, 36]. HCWs also recognized how problems with transportation impacted women’s access to ANC and were more focused on such problems during times of emergencies [42]. HCWs described situations in which women in critical condition were transported to the hospital via “boda-boda” due to the inability to obtain proper services [42].

### Occupation and male support

Both women and men described the influence of occupation on their ability to attend ANC. For women, their duties in the household prevented regular attendance; household work, childcare, and other responsibilities had to be completed before attending ANC [38, 43]. Transportation time and anticipated long hours spent at health facilities dissuaded women from attending ANC as they would lose working hours [38]. The same was true for women with formal employment.

Men placed emphasis on their occupation and financial gain to a greater extent. Men believed that attending ANC with their partners cost them time. This time could have been used to earn money for the well-being of their partner and future child [35, 43, 45]. With the additional financial strain of pregnancy and health care-related costs, male partners often had no choice but to attend work rather than accompany partners to ANC [34, 45, 46]. While some men understood the importance of ANC, they felt their overall responsibility lay in the financial aspects of pregnancy rather than attending ANC with their partners [43].

### Healthcare costs

While healthcare services were free of charge, supplemental costs of ANC in health facilities were found to be deterrents for early and regular pregnancy care [22, 36, 39]. For instance, women noted several HCWs charging for services provided and the use of bribes to be seen by HCWs faster and with higher quality service [38]. Furthermore, supplies such as “mama kits” and medications were not always available at all health facilities. In such cases, patients were required to procure and bring items to the health facility [39, 46, 47]. Some women noted that they were fearful of visiting health facilities without requested supplies as they would not be prioritized by HCWs, feel inferior, and not receive adequate care [46, 48].

### Cultural beliefs

#### Pregnancy perceptions

Responsibility of pregnancy almost always landed on the shoulders of women [40, 47, 49]. While some men took on the financial responsibility of paying for transportation, necessary equipment, and nutritious foods, others believed these to be the realm of “women’s money”, or money saved by women for childbirth and preparation [49, 50].

While increased community education has changed perceptions, some traditional views on pregnancy continued to propagate. For instance, both women and HCWs described women’s interpretation of pregnancy as a healthy state: that is, pregnancy is a normal and expected outcome of female life in which ANC or other forms of healthcare are unnecessary until complications arise [36, 47]. If needed, women knew to obtain advice from older women in their community or local TBAs [22, 35, 36, 47]. These beliefs hindered women’s access to early and regular ANC. HCWs also noticed that pregnant women tended to rely on older women in the community for information, especially if women had not experienced any pregnancy-related health risks in the past [36]. In some communities, pregnancy was not acknowledged by either woman or community until her “stomach” was visible [38, 47, 51]. This was to prevent the effects of “evil spirits” and those wishing poorly on the mother [38]. Some community health workers (CHWs) noted difficulties in identifying pregnant women and therefore initiating timely ANC [51]. While misconceptions have decreased in general, stigmatization has caused many teenage and older pregnant women to continue concealing pregnancy [41].

#### Traditional medicine

The use of traditional medicine, and more specifically TBAs, was recognized as a potential influencing factor on the uptake of ANC and the interaction between the patient and provider. Some HCWs admitted that they did not believe cultural beliefs interfered with ANC [35], felt confident in TBA’s ability to refer women to the health facility if necessary [52], and were aware that several TBAs advised early and regular ANC [35]. TBAs are not licensed to practice by the government of Uganda [52] but offer women a cheaper and closer alternative to health facilities [34, 42, 47] and provide more birthing options for women [52]. From the literature reviewed, TBAs were seen as respected sources of health information, in which women, men, and elders held in high confidence [42, 47, 52]. TBAs also possessed the unique ability to supply pregnant women with power and decision-making in the face of opposing male partners, as discussed below. While TBAs hold potential for pregnancy care, there is little to no supervision of such practice and no regulated training.

### Lack of autonomy and gender nuances

Themes of women’s empowerment and autonomy persisted within the literature. Lack of female autonomy led to decreased ANC initiation and retention [22, 35, 37, 38, 40, 47]. With little access to money, studies described unilateral decision making by women as a “problem” [49]. Women in many studies recounted the need for permission from their husbands to attend ANC and pay for necessary ANC supplies and transportation to the health facility [34, 35, 37, 49, 53].

Much of the power dynamics stemmed from financial dependence and cultural expectations. Women who were denied financial support from their spouses were unable to afford transportation to and from ANC (see *Transportation)*. Relationship insecurity for pregnant mothers further influenced power dynamics as some women were distrusting of their partners, feared breakups, neglect and domestic violence [38]. Polygamy and infidelity, seen as cultural norms in some communities, further strained pregnancy responsibility, leaving men with limited time to attend ANC, and economic resources available to women [45]. In one study, men agreed to comply with ANC guidelines and avoid infidelity if their pregnant partners would not withhold from sexual relations [45]. Such statements were seen by both women and HCWs to perpetuate gender inequity [45]. Among HCWs, gender dynamics were noted to impact ANC both positively and negatively. For instance, women received healthcare faster with a man present, as to respect men’s time [36, 44], and HCWs found pregnancy counselling and decision making easier with male presence [43, 45]. However, HCWs also reported limited ability of the woman to advocate for herself and ask questions in the presence of male companions [36]. At times, HCWs and women found it difficult to explain the importance of ANC to partners [34, 38, 43, 47, 50, 54] as men described their priority to be the future child’s health [49, 50, 55]. Nevertheless, both HCWs and women described the unique ability of TBAs as an intermediary for women and men [52]. As male partners were the main providers of financial resources, the ultimate ability to attend ANC was often a man’s decision [34, 35, 37, 49, 53]. TBAs, as respected women in the community, were able to persuade men of ANC’s importance [52]. One study noted that TBAs worked with women whose male partners refused ANC by charging “more” and providing women with the excess money to attend ANC [52].

While pregnancy responsibility lay in the hands of women, some men found it stigmatizing to be seen accompanying their partners to ANC. For instance, HCWs confirmed the notion that in many communities, men who attended ANC were considered “idle and weak” [45]. Overall, reasons for male refusal of ANC included lack of knowledge of the importance of ANC, lack of financial resources, fear of testing for HIV and other sexually transmitted infections (especially for men with multiple wives or partners), and cultural norms surrounding pregnancy care [45].

### Quality of ANC

The perceived quality of care influenced both attendance and retention in ANC. Three major themes emerged from the data: health facility resources, the presence of competent providers, and the interactions between patients and their providers.

#### Health facility resources: staff and resource shortages

Women were often met with large delays in care due to lack of staff [33, 38, 45, 47, 48, 56, 57]. When care was eventually received, women frequently described it as inadequate and of low quality including missed steps or education. HCWs reported feeling overburdened and ascribed such practices to extreme workloads and high patient numbers [48, 58]. As information such as long wait times and perceived low-quality care circulated in society, women became wary of health facilities, further decreasing ANC attendance [47, 59]. Simultaneously, the anticipated workload has led to poor recruitment and retention of HCWs, further perpetuating difficulties with human resources [57].

Both women and HCWs referenced an almost constant shortage of medical resources [35, 48]. Access to water, electricity, sanitation, bed space and equipment were not readily available in health facilities [42, 57–59]. During regular ANC, HCWs noted a lack of pregnancy tests, reagent strips, stethoscopes, fetal scopes, urine tests, and anemia tests, which led to gaps in adequate ANC [42]. Women and HCWs also noted a lack of emergency services [42, 58]: HCWs confirmed a lack of emergency obstetric care. For instance, women were referred to district hospitals with no assured transportation [51, 58]. Women also noted that lifesaving services, such as skilled birthing attendants at birth or emergency obstetric care was not offered 24 h a day [42, 58]. This insufficiency in medical resources placed a large strain on women’s trust and confidence in health facilities. As women grew aware of the insufficient drug supply, equipment, and health workers, as well as the long wait times and poor opening hours, some chose to access services from the community, such as TBAs, or remain home until delivery.

#### Competent providers

Much of what pregnant women and family members described as good quality ANC referred to being seen by competent providers; providers who were adequately able to educate and communicate, provide treatment, and solve issues.

### Pregnancy education

The literature pointed to a deficit in pregnancy education. While pregnancy education was described by women as an important reason to attend ANC [35, 51], it was not always provided. Much of the education took place in group settings rather than individual ANC visits [44, 58]. These sessions were moderated by midwives and addressed different topics each day [58]. However, the lack of a systematic education system [58] made it difficult to ensure all pregnant women had received proper and adequate education. For instance, women described a low level of knowledge on topics such as nutrition [44, 58], danger signs of pregnancy [44, 58], postpartum depression, and HIV transmission [44]. They also noted insufficient information provided on medications received [58] and on tests and procedures. Some women obtained information from other women in line for ANC services [44, 56]. In one study, women saw the lack of knowledge as a fault of their own, as this was their responsibility rather than HCWs [58].

From male partner’s perspectives, pregnancy education felt lacking in male specific initiatives and programs, which caused them to lose motivation for involvement. From the HCW’s perspective, they believed education to be lacking due to women’s absence from morning sessions [18, 56], staff shortages [18, 42, 58, 60], and lack of community outreach [35, 44, 48, 51, 57]. HCWs called for increased community involvement in education efforts, rather than sole reliance on health facilities [44].

### Provider qualifications

One tenant of good quality care came by way of a knowledgeable provider; women in this context considered their ANC of “good quality” when they were received by qualified and well-trained staff [59]. Many women believed such training extended from medicine into interpersonal relationships, attitude, and communication [59]. However, due to a lack of staffing and a high patient: provider ratio [59], HCWs admitted to staff quality dropping [54]. For instance, overburdened staff drove facilities to hire unqualified nursing assistants [42], decreasing the quality of healthcare received. This review did not cover the breadth or caliber of HCWs knowledge and education.

CHWs served as another source of information for many pregnant women in Uganda. These individuals, however, were seen to vary in ability and therefore trust in the community [51, 54]. For instance, due to the shortage of CHWs, not all met the national criteria of literacy [50, 54], which caused a level of mistrust and lack of confidence in their medical abilities within the community [50, 51]. However, both women and their families noted that with time and experience, they came to highly rely on CHWs [50]. Furthermore, HCWs in one study considered CHWs technically very strong and an important source of knowledge and skills in the community [51].

TBAs additionally served as another form of care provider, while not sanctioned by the Ugandan government. Some family members and women saw TBAs as less risky due to the presence of unqualified nursing assistants in health facilities, as well as facility shortages of drugs and supplies during delivery [42]. See *Cultural Beliefs* for further information pertaining to TBAs.

#### Patient-provider interactions

The psychosocial aspects of healthcare were described as of equal importance to areas of care such as infrastructure and clinical medicine. This included respect, privacy, trust, and communication. Women desired to be treated with empathy and compassion and have their decision-making process respected [59]. Throughout the reviewed studies, poor attitudes and actions perpetuated by HCWs left some women feeling detached from their own health and a tendency to avoid ANC altogether [35, 47, 48, 58]. HCW’s attitudes served as a reason for non-enrollment in ANC [22, 35, 38, 39] and news of such behaviors circulated through the communities. Women expected low quality care [48]. Numerous papers recount abuse towards pregnant women including neglect, mistreatment, and refusal to treat [22, 46–48]. Women in vulnerable populations recounted increased discrimination. This included women of high parity [48], late ANC bookers [38], women living in poverty [38, 39, 48], adolescents [39, 41], or previous use of TBAs and herbal medicine [48]. Parents of pregnant teenagers described the blame and shame HCWs placed on their children as well as longer wait times compared to other patients [41]. Many teenage patients already lacked support from their families or the fathers of children [41] and such reactions from HCWs, together with increased stigmatization, pushed these adolescents further from the healthcare system. Male partners also noticed the lack of respect from HCWs [42] leading some to prefer treatment by TBAs [52]. Such behaviors by HCWs decreased male participation and ANC attendance [43, 45, 55].

The lack of adequate communication and sensitization was found to be a major factor for poor use of maternal health services [42]. Women described good provider communication as using clear, concise, and positive language with words that were easily understood and with minimal medical terminology [59]. Women desired for HCWs to listen to their needs while building rapport between HCWs and the woman herself and her family to enable a relationship based on trust [59]. Additionally, women wanted privacy when receiving care [38, 48]. For instance, ANC counseling or initial ANC assessment was often provided in front of other patients [59]. Although a major barrier to improvement is the lack of facility space [45, 48], some HCWs recounted that the situation could be better if the right to privacy is a priority for HCWs [48, 55].

In some instances, HCWs recognized their attitudes and the effects on ANC provision. HCWs claimed such actions were due to lack of time and room for privacy [48]. Nevertheless, in certain instances where women recounted disrespect, abuse, and neglect from HCWs, such providers were oblivious to problems [47]. In fact, some HCWs believed women deliberately came to ANC late to avoid educational topics [56], and therefore merited worse treatment.

### Strategies for increasing ANC accessibility

Factors which increased accessibility and feasibility of quality ANC were also recognized within the literature, such as strategies for positive change and the implementation of services to enhance experiences with ANC, improve adherence, and the timeliness of visits.

Included studies have explored methods to increase female autonomy and empowerment, for instance, the use of saving groups [46, 49, 50]. Women’s saving groups consist of a collective of women who come together to save money and provide financial assistance to one another, with the aim of expanding women’s access to financial resources when needed [46]. Such groups also allowed women to better navigate power balances between male partners, as they were no longer reliant on men’s financial support and decision making. Additionally, employment or full-time occupation was seen to improve women’s access to ANC due to similar reasons [46].

With inadequate clinic resources, HCWs, and medications, women found it difficult to justify the needs and benefits of ANC, with some women choosing other providers or facilities (private clinics, TBAS, etc.) instead [42, 45, 47, 48]. Women have proposed strategies for improvement to access and quality of ANC. For instance, women called for improved supply of drugs and equipment, and better workforce, especially in lower-level facilities [58], as women had higher accessibility to peripheral facilities [58]. Similarly, men desired shorter wait times in clinics through improvements in efficiency and strengthened efforts in privacy and confidentiality [43]. Lastly, both men and women insisted that changes in provider attitudes and interactions with patients would make ANC more enjoyable and accessible [43, 49].

The necessity for improved education and shared responsibility became clear through this review. With no uniform pregnancy education system in place, the risk of missed and lacking education was high and fell solely on the shoulders of women [58]. For that reason, both women and men discussed strategies to promote and improve information sharing during ANC [46]. Women suggested the need for conversation promoting partnership between partners and encouraging men to join educational ANC visits [49]. Women also desired better incorporation of men in maternal and reproductive health to reduce gender inequities in the community [42]. Men engaged with these topics more concretely, calling for community-based communication approaches to educate a greater number of men about maternal health, for instance increasing door-to-door mobilization, media campaigns (radio, print), community seminars, and further development of mobile services [43–45].

## Discussion

We reviewed and synthesized the qualitative and quantitative literature on the perceptions and attitudes of pregnant women, their families, and HCWs towards ANC as well as the main factors affecting ANC uptake in Uganda. Our findings underscore the significance of socio-demographic determinants such as maternal education, wealth, and marital status, demographic and obstetric factors like maternal age, regional disparities, and parity. Our results also elucidate the critical role of financial and cultural barriers, including transportation costs and local health beliefs. Overall, this review offers insights for shaping evidence-based interventions to enhance ANC access and quality, and such strategies and interventions will be the focus of this discussion.

Critical shortfalls in ANC provision are pregnancy information, health education and provider attitudes; key areas where ANC quality may be improved [8, 9]. Offering ANC to women based on age and gestational age can improve this aspect of service quality and we found a possible association between women’s age and ANC uptake [44]. This benefit can also be seen through the enhancement of age and gestational age-specific group learning [46], to maintain [18] and provide clients with confidence in their healthcare. Akin to pregnancy education by gestational age, other intervention packages can be tailored [46] to improve efficiency in clinical workflow and procedures. Examples include shorter wait times [43], wider health facility opening hours [35], improved privacy and confidentiality [43], and more numerous HCWs and human resources [38]. Screening for women that book late or those with markers of social vulnerability (relationship insecurity, unwanted pregnancy) and providing targeted support may ultimately allow facilitated access to ANC [38]. The lack of routine ways to measure and monitor quality of care and effects of such interventions should also be addressed. Seemingly obvious, yet economically challenging, are improvements like better infrastructure, digitized records, and supplies [58]. Any health system intervention is reliant on overall improvements in service provision in the public health sector [18].

Cultural and societal factors play an important role in pregnancy care and outcomes. Much of this stems from power and gender imbalances within societies that trickle into ANC. Innovations that aim to increase early and regular ANC must target autonomy and decision-making processes in families and communities. Increased dialogue about the importance of shared decision making and the image of partnership between men and women must be supported [49], either through media campaigns, community education campaigns, or the involvement of local leaders. Such leaders may also act as an entry point into communities to communicate the importance and advantages of male partner involvement, financial saving, and birth preparedness [50]. Further empowerment of women may come in the form of saving groups [46, 50], which may have similar forms of information dissemination, or female financial independence [36, 40, 46, 50] via paid employment. Male involvement in ANC can be strengthened by peer engagement. For instance, the promotion of ANC attendance by other fathers or male partners within clinics [43], personal invitations for male partners to visit the clinic with women [43], or the use of visual tools by male CHWs to educate and engage men in the pregnancy process [46]. As such strategies and innovations develop, so should the promotion of participatory health systems [48]. The emphasis within health clinics should be shifted from paternal forms of leadership in health to spaces for equitable patient-centered healthcare [48]. Allocating resources to strengthen midwifery practice and midwife led community care could improve client centeredness [47].

Several studies mentioned the role of TBAs in providing culturally appropriate ANC. The position of TBAs within communities, as older, knowledgeable, trusted women, lands them in a place of power and respect. The studies discussed how some women visited TBAs rather than health facilities for ANC and delivery. A formal collaboration with TBAs may serve as a successful innovation to improve maternal and neonatal health outcomes, especially in rural communities. For instance, training TBAs in biomedical knowledge and skills may allow ANC information and pregnancy education to disseminate further into the communities [52]. However, at present, the use of TBAs for maternal healthcare is not supported by the Ugandan Ministry of Health (MOH).

Improving transportation infrastructure and availability can make ANC services more accessible, especially in remote areas [33–39]. In the presence of geographic barriers, improvement of community-based ANC, such as mobile clinics or outreach program, can increase ANC accessibility [8]. Many studies also discussed the role of CHWs in providing community-based ANC. CHWs could play a vital part in mobilizing women and families to attend ANC at health facilities, for example through door-to-door visits, community gatherings, and group education [51, 54]. While ANC in Uganda is expected to be provided free-of-charge, many women describe the additional costs incurred at health clinics. Our findings showed a clear positive association between wealth and ANC uptake. Interventions such as providing financial assistance for ANC can help alleviate the financial burden on pregnant women and their families and incentivize ANC utilization [7, 61].

As Uganda begins the implementation of 8, rather than 4, ANC contacts, as recommended by the WHO, several challenges will arise. This review was conducted as part of an implementation research project for this transition and will inform the Uganda MOH’s plans for the new guideline rollout. Doubling the number of ANC contacts is likely to bring severe constraints on already limited human resources and health systems. If no measures for equitable implementation are taken, increased ANC visits may result in increased clinic traffic, prolong wait times, and hamper the quality of ANC provided. The review describes the current shortage in medical equipment and medical supplies and the numerous barriers women recount to attending ANC. Women require money for transportation, lose money waiting for care, and they often pay out of pocket for supplies. Policymakers should be aware of these challenges when planning for the implementation of 8 ANC contacts.

### Strengths and limitations

This is the first review to synthesize evidence focused on barriers and facilitators to ANC services from the perspective of these three distinct stakeholders in Uganda. Qualitative studies were included as they provide in-depth insights into complex social phenomena and provide rich data on the attitudes, beliefs, and experiences of individuals. Such studies allowed for a thorough exploration of experiences, cultural and social norms, and contextual factors that influence women’s utilization of ANC. This information can support the development of culturally sensitive and informed interventions aimed at improvements in this field. Quantitative studies were included as they allow examination of relationships between variables, comparisons across groups, and identification of patterns. These studies provided information on the distribution of socioeconomic and lifestyle characteristics, as well as behaviors towards ANC.

Individual qualitative studies were assessed for methodological limitations based on the Critical Appraisal Skills Programme (CASP) [14] checklist. However, we did not assess how the methodological limitations in the studies may have influenced the confidence in the findings reported in this review. Another limitation is the data used in the studies included in the quantitative analysis as almost all studies included used data from the DHS and therefore carried with it the inherent limitations of this data source.

## Conclusion

This systematic review has examined the diverse factors influencing antenatal care utilization in Uganda, considering the perspectives of women, their families, and healthcare workers. Our findings emphasize the need to address healthcare quality concerns, including staff shortages and deficiencies in pregnancy education, as well as negative patient-provider interactions. Targeted initiatives are essential to bridge existing gaps and ensure equitable ANC access for all women in the country.

## Electronic supplementary material

Below is the link to the electronic supplementary material.

Additional file 1. Search strategy

Additional file 2: Critical Appraisal Skills Programme (CASP) Checklist

Additional file 3: List of Included Studies

## Data Availability

The datasets supporting the conclusions of this article are referenced within the article and can be provided upon request.

## Abbreviations

ANC: Antenatal Care
ANC8: 8 ANC contacts as recommended by the World Health Organization
CASP: Critical Appraisal Skills Programme
CHW: Community health Worker
DHS: Demographic and Health Survey
HC: Health center
HCW: Healthcare workers
LMIC: Low-middle income country
MOH: Ministry of Health
TBA: Traditional birthing attendant
VHT: Village health team
WHO: World Health Organization
