# Correction: Factors influencing the uptake of antenatal care in Uganda: a mixed methods systematic review

**DOI:** 10.1186/s12884-024-07001-0

**Published:** 2024-11-27

**Authors:** Kiran Bhutada, Mahima Venkateswaran, Maureen Atim, Susan Munabi-Babigumira, Victoria Nankabirwa, Flavia Namagembe, J. Frederik Frøen, Eleni Papadopoulou

**Affiliations:** 1 Global Health Cluster, Division for Health Services, Norwegian Institute of Public Health, PO Box 222, Skøyen, Oslo Norway; 2 Global Health Center, Albert Einstein College of Medicine, 1300 Morris Park Avenue Block 505, Bronx, NY 10461 USA; 3 Centre for Intervention Science in Maternal and Child Health (CISMAC), University of Bergen, Bergen, Norway; 4 Makerere University School of Public Health, New Mulago Gate Rd, Kampala, Uganda; 5 Department of Health and Functioning, Western Norway University of Applied Sciences, Bergen, Norway

**Correction: BMC Pregnancy Childbirth 24**,** 730 (2024)**

10.1186/s12884-024-06938-6

Following publication of the original article [1], the authors reported an error in Author’s affiliations. Affiliations are correct but the allocation of affiliation per author is not correct.

The author group has been updated above and the original article [1] has been corrected.
