# Supplementary material for: Factors influencing the uptake of antenatal care in Uganda: a mixed methods systematic review
Source: BMC Pregnancy Childbirth. 2024 Nov 8;24:730. doi: 10.1186/s12884-024-06938-6 (PMC11545493; doi:10.1186/s12884-024-06938-6)
Supplement: Supplementary file 1 — Additional file 1. Search strategy [file 12884_2024_6938_MOESM1_ESM.docx]

Search strategies

**Ovid MEDLINE(R) ALL <2012 to September 07, 2022> (searched 08. September 2022)**

| **No.** | **Search terms** | **Results** |
| --- | --- | --- |
| 1 | Prenatal Care/ | 31488 |
| 2 | Perinatal Care/ | 5228 |
| 3 | Pregnant Women/ | 12789 |
| 4 | Delivery of Health Care/ | 110334 |
| 5 | 3 and 4 | 165 |
| 6 | ((antenatal or ante natal or prenatal or pre natal or perinatal or peri natal or antepartum or ante partum or pregnancy) and (care or checkup? or check up? or service? or support or visit?)).ti. | 11437 |
| 7 | ((antenatal or ante natal or prenatal or pre natal or perinatal or peri natal or antepartum or ante partum or pregnancy) adj3 (care or checkup? or check up? or service? or support or visit?)).ab,kf. | 37271 |
| 8 | 1 or 2 or 5 or 6 or 7 [ANC] | 63723 |
| 9 | uganda*.ti,ab,kf,hw. | 20642 |
| 10 | Qualitative Research/ | 76320 |
| 11 | Narration/ | 9768 |
| 12 | Interviews as Topic/ | 66803 |
| 13 | Focus Groups/ | 34697 |
| 14 | Surveys and Questionnaires/ | 543419 |
| 15 | (focus group* or qualitative or ethnograph* or fieldwork or field work or key informant or interview* or mixed method? or survey*).ti,ab,kf. | 1346926 |
| 16 | ((semi structured or semistructured or unstructured or informal or in depth or indepth or face to face or structured or guide*) adj3 (discussion* or questionnaire*)).ti,ab,kf. | 36603 |
| 17 | 10 or 11 or 12 or 13 or 14 or 15 or 16 [Based on a Qualitative filter from University of Texas] | 1692179 |
| 18 | 8 and 9 and 17 | 317 |
| 19 | limit 18 to yr="2012 -Current" | 255 |

**Embase <1974 to 2022 September 07> (searched 08. September 2022)**

| No. | Search terms | Results |
| --- | --- | --- |
| 1 | prenatal care/ | 46403 |
| 2 | perinatal care/ | 15415 |
| 3 | pregnant woman/ | 101088 |
| 4 | health care delivery/ | 197700 |
| 5 | 3 and 4 | 1043 |
| 6 | ((antenatal or ante natal or prenatal or pre natal or perinatal or peri natal or antepartum or ante partum or pregnancy) and (care or checkup? or check up? or service? or support or visit?)).ti. | 13582 |
| 7 | ((antenatal or ante natal or prenatal or pre natal or perinatal or peri natal or antepartum or ante partum or pregnancy) adj3 (care or checkup? or check up? or service? or support or visit?)).ab,kf. | 47408 |
| 8 | 1 or 2 or 5 or 6 or 7 [ANC] | 88001 |
| 9 | Uganda/ | 19707 |
| 10 | uganda*.ti,ab,kf. | 21941 |
| 11 | 9 or 10 | 24082 |
| 12 | qualitative research/ | 104206 |
| 13 | exp interview/ | 339412 |
| 14 | questionnaire/ | 829163 |
| 15 | (focus group* or qualitative or ethnograph* or fieldwork or field work or key informant or interview* or mixed method? or survey*).ti,ab,kf. | 1697166 |
| 16 | ((semi structured or semistructured or unstructured or informal or in depth or indepth or face to face or structured or guide*) adj3 (discussion* or questionnaire*)).ti,ab,kf. | 49676 |
| 17 | 12 or 13 or 14 or 15 or 16 | 2309490 |
| 18 | 8 and 11 and 17 | 381 |
| 19 | limit 18 to (embase and yr="2012 -Current") | 170 |

**Cinahl**

| S24 | S21 AND S22 [Limiters - Exclude MEDLINE records] | 58 |
| --- | --- | --- |
| S23 | S21 AND S22 | 112 |
| S22 | EM 2012- | 4,357,599 |
| S21 | S4 AND S7 AND S20 | 185 |
| **S20** | **S8 OR S9 OR S10 OR S11 OR S12 OR S13 OR S14 OR S15 OR S16 OR S17 OR S18 OR S19** | 1,078,144 |
| S19 | TI ( (semi-structured or semistructured or unstructured or informal or in-depth or indepth or face-to-face or structured or guide*) W3 (discussion* or questionnaire*) ) OR AB ( (semi-structured or semistructured or unstructured or informal or in-depth or indepth or face-to-face or structured or guide*) W3 (discussion* or questionnaire*) ) | 15.124 |
| S18 | TI ( (focus-group* or qualitative or ethnograph* or fieldwork or field-work or key-informant or interview* or mixed-method* or survey*) ) OR AB ( (focus-group* or qualitative or ethnograph* or fieldwork or field-work or key-informant or interview* or mixed-method* or survey*) ) | 633.453 |
| S17 | (MH "Narrative Medicine") | 124 |
| S16 | (MH "Structured Questionnaires") | 8.31 |
| S15 | (MH "Questionnaires") | 456.74 |
| S14 | (MH "Surveys") | 159.28 |
| S13 | (MH "Focus Groups") | 49.082 |
| S12 | (MH "Delphi Technique") | 7.484 |
| S11 | (MH "Structured Interview") | 10.696 |
| S10 | (MH "Semi-Structured Interview") | 74.862 |
| S9 | (MH "Interviews") | 162.103 |
| S8 | (MH "Qualitative Studies+") | 172.846 |
| **S7** | **S5 OR S6** | 7.435 |
| S6 | TI uganda* OR AB uganda* | 6.631 |
| S5 | (MH "Uganda") | 5.856 |
| **S4** | **S1 OR S2 OR S3** | 33.789 |
| S3 | TI ( (antenatal or ante-natal or prenatal or pre-natal or perinatal or peri-natal or antepartum or ante-partum or pregnancy) W3 (care or checkup* or check-up* or service* or support or visit*) ) OR AB ( (antenatal or ante-natal or prenatal or pre-natal or perinatal or peri-natal or antepartum or ante-partum or pregnancy) W3 (care or checkup* or check-up* or service* or support or visit*) ) | 18.498 |
| S2 | (MH "Perinatal Care") | 5.109 |
| S1 | (MH "Prenatal Care") | 19.203 |

**Scopus, Elsevier (searched 08. September 2022)**

| **No.** | **Search Terms** | **Results** |
| --- | --- | --- |
| 1 | ( TITLE-ABS-KEY ( antenatal OR "ante natal" OR prenatal OR "pre natal" OR perinatal OR "peri natal" OR antepartum OR "ante partum" OR pregnancy ) PRE/3 ( care OR checkup* OR "check up" OR "check ups" OR service* OR support OR visit* ) ) AND ( TITLE-ABS-KEY ( uganda* ) ) AND ( TITLE-ABS-KEY ( "focus group" OR "focus groups" OR qualitative OR ethnograph* OR fieldwork OR "field work" OR "key informant" OR interview* OR "mixed method" OR "mixed methods" OR survey* OR "semi structured" OR semistructured OR unstructured OR informal OR "in depth" OR indepth OR "face to face" OR "structured discussion" OR "structured discussions" OR "structured questionnaire" OR "structured questionnaires" ) ) AND NOT INDEX ( medline ) | 49 |

**Global Index Medicus, WHO:** [**https://www.globalindexmedicus.net/**](https://www.globalindexmedicus.net/) **(searched 08. September 2022)**

| No. | Search terms | Results |
| --- | --- | --- |
| 1 | tw:((tw:(antenatal OR "ante natal" OR prenatal OR "pre natal" OR perinatal OR "peri natal" OR antepartum OR "ante partum" OR pregnancy )) AND (tw:(uganda*)) AND (tw:("focus group" OR "focus groups" qualitative OR ethnograph* OR fieldwork OR "field work" OR "key informant" OR interview* OR "mixed method" OR "mixed methods" OR survey* OR "semi structured" OR semistructured OR unstructured OR informal OR "in depth" OR indepth OR "face to face" OR "structured discussion" OR "structured discussions" OR "structured questionnaire" OR "structured questionnaires" ))) | 26 |

**Epistemonikos, Epistemonikos Foundation:** [**https://www.epistemonikos.org/**](https://www.epistemonikos.org/) **(searched 08. September 2022)**

| 1 | Title/Abstract: (antenatal OR "ante natal" OR ante-natal OR prenatal OR "pre natal" OR pre-natal OR perinatal OR "peri natal" OR peri-natal OR antepartum OR "ante partum" OR ante-partum OR pregnancy) AND (care OR checkup* OR "check up" OR "check ups" OR check-up OR check-ups OR service* OR support OR visit*) |  |
| --- | --- | --- |
|  | AND |  |
| 2 | Title/Abstract: uganda* |  |
|  | AND |  |
| 3 | Title/Abstract: "focus group" OR "focus groups" OR focus-group OR focus-groups OR qualitative OR ethnograph* OR fieldwork OR "field work" OR field-work "key informant" OR key-informant OR interview* OR "mixed method" OR "mixed methods" OR mixed-method OR mixed-methods OR survey* OR semistructured OR "semi structured" OR semi-structured OR unstructured OR informal OR indepth OR "in depth" OR in-depth OR "face to face" OR face-to-face OR "structured discussion" OR "structured discussions" OR "structured questionnaire" OR "structured questionnaires" | 167 |
