# Supplementary material for: Factors influencing the uptake of antenatal care in Uganda: a mixed methods systematic review
Source: BMC Pregnancy Childbirth. 2024 Nov 8;24:730. doi: 10.1186/s12884-024-06938-6 (PMC11545493; doi:10.1186/s12884-024-06938-6)
Supplement: Supplementary file 2 — Additional file 2: Critical Appraisal Skills Programme (CASP) Checklist [file 12884_2024_6938_MOESM2_ESM.docx]

# Critical Appraisal Skills Programme Checklist (CASP)

| **Study ID** | **Was there a clear statement of the aims of research?** | **Is it qualitative methodology appropriate?** | **Was the research design appropriate to the aims of the research?** | **Was the recruitment strategy appropriate to the aims of the research?** | **Was the data collected in a way that addressed the research issue** | **Has the relationship between researcher and participants been adequately considered?** | **Have ethical issues been taken into consideration?** | **Was the data analysis sufficiently rigorous?** | **Is there a clear statement of findings?** | **How valuable is the research?** | **Methodological Limitations present?** |
| --- | --- | --- | --- | --- | --- | --- | --- | --- | --- | --- | --- |
| Bohren 2017 | Yes | Yes | Yes | Yes | Yes | No | Yes | Yes | Can't tell | Valuable |  |
| Chi 2015 | Yes | Yes | Yes | Yes | Yes | No | Yes | Yes | Yes | Can't tell |  |
| Conrad 2012 | Yes | Yes | Yes | Yes | Yes | No | Yes | Yes | Yes | Valuable |  |
| Tetui 2012 | Yes | Yes | No | Yes | No | No | Yes | Can't tell | Yes | Can't tell | Yes |
| Lubega 2013 | Yes | Yes | No | Yes | Yes | Yes | Yes | Yes | Yes | Valuable |  |
| Ayiasi 2013 | Yes | Yes | Yes | Yes | Yes | No | Yes | Yes | Yes | Valuable |  |
| Atekyereza 2014 | Yes | Yes | Yes | Yes | Yes | No | Yes | No | No | Valuable | Yes |
| Okuga 2015 | Yes | Yes | Can't Tell | Can’t Tell | Yes | No | No | No | Yes | Can't tell | Yes |
| Turinawe 2016 | Yes | Yes | Yes | Yes | No | Yes | Yes | Yes | Yes | Valuable | Yes |
| Ekirapa-Kiracho 2016 | Yes | Yes | Yes | Yes | Yes | No | Yes | Yes | Yes | Valuable |  |
| Sileo 2017 | Yes | Yes | Yes | Yes | Yes | No | Yes | Can't tell | Yes | Valuable |  |
| Sarkar 2018 | Yes | Yes | Can't Tell | Yes | Yes | Yes | Yes | Yes | Yes | Valuable |  |
| Rukundo 2019 | Yes | Yes | Can't Tell | Can't tell | Can't tell | No | Yes | Can't tell | Yes | Valuable | Yes |
| Wilson 2019 | Yes | Yes | Yes | No | Yes | No | Yes | No | Yes | Can't tell | Yes |
| Uldbjerg 2020 | Yes | Yes | Yes | Yes | Yes | No | Yes | Yes | Yes | Valuable |  |
| Steele 2021 | Yes | Yes | Can't Tell | Can't tell | No | No | Yes | Yes | Yes | Valuable | Yes |
| Roed 2021 | Yes | Yes | Yes | Yes | Yes | No | Yes | Yes | Yes | Valuable |  |
| Alhassan 2022 | Yes | Yes | Yes | Yes | Yes | No | Can't tell | Yes | Yes | Valuable |  |
| NambileCumber 2022 | Yes | Yes | Yes | Yes | Yes | Yes | Yes | No | Yes | Valuable |  |
