# Supplementary material for: Factors influencing the uptake of antenatal care in Uganda: a mixed methods systematic review
Source: BMC Pregnancy Childbirth. 2024 Nov 8;24:730. doi: 10.1186/s12884-024-06938-6 (PMC11545493; doi:10.1186/s12884-024-06938-6)
Supplement: Supplementary file 3 — Additional file 3: List of Included Studies [file 12884_2024_6938_MOESM3_ESM.docx]

# List of Included Studies

| **Study ID** | **Study design** | **Geographic area** | **Context** | **Population** | **Outcome** |
| --- | --- | --- | --- | --- | --- |
| Kalule-Sabiti 2014 | Quantitative | Uganda | Rural & urban | Women ; Family members | 4+ ANC visits |
| Singh 2014 | Mixed methods | Uganda | Rural | Women ; Family members | gender, finances, cultural beliefs |
| Sileo 2017 | Qualitative | Uganda | Rural | Women ; Family members | patient-provider relationship, gender, finances, quality of care |
| Muheirwe 2019 | Mixed methods | Uganda | Rural | Women ; Family members | gender, media access, patient-provider relationship, pregnancy education |
| Nyakato 2020 | Mixed methods | Uganda | Rural | Women ; Family members | 1+ ANC visits, first trimester ANC, gender, finances |
| Adjiwanou 2014 | Quantitative | Uganda; Ghana, Kenya, Tanzania | Rural | Women | gender |
| Agaba 2021 | Quantitative | Uganda | Rural & urban | Women | ANC in first trimester, 4+ ANC visits |
| Alhassan 2022 | Qualitative | Uganda;  South Africa | urban | Women | finances, pregnancy education, gender, patient-provider relationship, quality of care, facility resources, cultural beliefs |
| Andegiorgish 2022 | Quantitative | Uganda; Ghana, Kenya, Malawi, Namibia, Rwanda, Senegal, Tanzania, Uganda, Zambia | Rural & urban | Women | 4+ ANC visits, type of ANC provider, first trimester ANC |
| Atekyereza 2014 | Qualitative | Uganda | Rural | Women ; Healthcare workers | age, gender, location, mother's education, cultural beliefs, first trimester ANC, previous pregnancy experience |
| Atuoye 2020 | Quantitative | Uganda | Rural & urban | Women | 4+ ANC visits, first trimester ANC |
| Ayiasi 2013 | Qualitative | Uganda | Rural | Women ; Healthcare workers | facility resources, pregnancy education, quality of care, patient-provider relationship |
| Babughirana 2020 | Quantitative | Uganda | Rural | Women | 4+ ANC visits, first trimester ANC, quality of care, facility resources |
| Banke-Thomas 2022 | Mixed methods | Uganda; Guinea, Nigeria, Tanzania | Rural & urban | Healthcare workers | COVID19, facility resources, competent providers, first trimester ANC |
| Bariagaber 2016 | Quantitative | Uganda | Rural & urban | Women | 4+ ANC visits |
| Belaid 2021 | Mixed methods | Uganda | Rural | Women ; Healthcare workers ; Family members | finances, facility resources, patient-provider relationship, gender |
| Bohren 2017 | Qualitative | Uganda, Nigeria | Urban | Women | quality of care, patient-provider relationship, competent providers, facility resources |
| Chi 2015 | Qualitative | Uganda, Burundi | Rural | Women ; Healthcare workers | COVID19, facility resources, competent providers, first trimester ANC |
| Conrad 2012 | Mixed methods | Uganda | Rural | Women ; Healthcare workers | quality of care, facility resources, emergency services, pregnancy education, patient-provider interaction |
| Delzer 2021 | Mixed methods | Uganda | Rural | Women ; Healthcare workers | pregnancy education, finances, cultural beliefs, age, gender |
| Ekirapa-Kiracho 2016 | Qualitative | Uganda | Rural | Women | pregnancy education, gender, finances, location, competent providers, facility resources |
| Ekirapa-Kiracho 2017 | Quantitative | Uganda | Rural | Women ; Healthcare workers ; Family members | first trimester ANC, 4+ ANC visits |
| Kananura 2017 | Mixed methods | Uganda | Rural | Women ; Healthcare workers ; Family members | 4+ ANC visits |
| Kananura 2017 | Quantitative | Uganda | Rural | Women | facility resources, competent providers, emergency resources, finances, location, pregnancy education |
| Kawungezi 2015 | Mixed methods | Uganda | Rural | Women ; Healthcare workers | quality of care, previous pregnancy experience, education, location, finances, occupation, ANC fear, cultural beliefs |
| Kayongo 2019 | Qualitative | Uganda | Rural & urban | Women ; Healthcare workers ; Family members | patient-provider relationship, media access, gender, fear, cultural beliefs, occupation, facility resources |
| Lubega 2013 | Qualitative | Uganda | Rural | Women ; Healthcare workers | transportation, ANC location, pregnancy education, facility resources |
| Namazzi 2017 | Mixed methods | Uganda | Rural & urban | Healthcare workers | competent providers, finances, quality of care, gender |
| Nambile Cumber 2022 | Qualitative | Uganda | Urban | Women | finances, location, patient-provider relationship, age, cultural beliefs |
| Okuga 2015 | Qualitative | Uganda | Rural & urban | Women ; Healthcare workers | gender, competent providers, cultural beliefs, patient-provider relationship, pregnancy education, facility resources |
| Roed 2021 | Qualitative | Uganda | Rural | Women ; Healthcare workers | pregnancy education, age, communication, finances, gender, patient-provider relationship, location |
| Rukundo 2019 | Qualitative | Uganda | Urban | Family members | age, pregnancy education, fear, competent providers, patient-provider relationship, finances, cultural beliefs |
| Saad-Haddad 2016 | Quantitative | Uganda, Bangladesh, Cambodia, Cameroon, Nepal, Peru, Senegal | Rural & urban | Women | ANC in first trimester, 4+ ANC visits, provision of ANC by skilled provider, quality of care, pregnancy education, location, mother's education |
| Sarkar 2018 | Qualitative | Uganda | Rural | Women ; Healthcare workers | patient-provider relationship, facility resources, quality of care, fear, cultural beliefs |
| Semaan 2022 | Mixed methods | Uganda, Guinea, Nigeria, Tanzania | Urban | Healthcare workers | lack of education, resources, patient-provider relationship |
| Serbanescu 2019 | Mixed methods | Uganda, Zambia | Rural & urban | Women ; Healthcare workers ; Family members | previous pregnancy experiences, pregnancy education |
| Sserwanja 2022 | Quantitative | Uganda | Rural & urban | Women | first trimester ANC |
| Sserwanja 2022 | Quantitative | Uganda | Rural & urban | Women | 4+ ANC visits, 8+ ANC visits, ANC in first trimester, type of ANC provider |
| Ssetaala 2020 | Quantitative | Uganda | Rural | Women | 4+ ANC visits, first trimester ANC |
| Steele 2021 | Mixed methods | Uganda | Rural | Women ; Healthcare workers | pregnancy education, location, finances, gender, patient-provider relationship, age |
| Tetui 2012 | Mixed methods | Uganda | Rural | Healthcare workers | facility resources, quality of care |
| Turinawe 2016 | Qualitative | Uganda | Rural | Healthcare workers ; Family members | competent providers, gender, patient-provider relationship |
| Turyasiima 2014 | Mixed methods | Uganda | Rural | Women ; Healthcare workers | cultural beliefs, previous pregnancy experience, gender, occupation, patient-provider relationship, pregnancy education, facility resources, finances |
| Uldbjerg 2020 | Qualitative | Uganda | Rural | Women ; Healthcare workers | facility resources, patient-provider relationship, cultural beliefs, pregnancy education, gender, age |
| Vogel 2016 | Mixed methods | Uganda, Myanmar, Tanzania, Ethiopia | Rural & urban | Women ; Healthcare workers ; Family members | facility resources, competent providers |
| Wilson 2019 | Qualitative | Uganda | Rural | Women ; Healthcare workers | facility resources, finances, pregnancy education, previous pregnancy experiences, cultural beliefs, gender, patient-provider relationship, |
| Workneh 2016 | Quantitative | Uganda, 31 Sub-Saharan African countries | Rural & urban | Women | 4+ ANC visits |
